# Boundary Vector Cells Encode a Future‐Biased Spectrum of Positions in the Rat

**DOI:** 10.1002/hipo.70110

**Published:** 2026-06-08

**Authors:** Ehren Lee Newman, Inna Mashanova‐Galikova, Zoran Tiganj, Colin Lever

**Affiliations:** ^1^ Department of Psychological and Brain Sciences Indiana University Bloomington Indiana USA; ^2^ Department of Computer Science Indiana University Bloomington Indiana USA; ^3^ Department of Psychology Durham University Durham UK

**Keywords:** navigation, rat, spatial tuning, subiculum, temporal tuning

## Abstract

Spatial tuning is a hallmark property of neural firing in the hippocampal formation. Yet, that tuning is often less well correlated with the instantaneous current position of an animal than it is with an integrated version of the past or future state of the animal. Whether that encoding is biased toward past or future states and the extent to which it shows fixed versus multi‐scale encoding varies across circuits and cell types. The temporal encoding properties of boundary vector cells of the subiculum are not well established. To address this here, we re‐analyzed recordings of boundary vector cells (BVCs) described previously by Lever et al. (2009) with multiple approaches. In the first, we asked if adding a temporal offset between the rat position and the spiking of a BVC increased the apparent spatial tuning in the firing rate map. We found that aligning BVC spiking with future states maximized the rate map spatial tuning. These results were mirrored in a second analysis that, instead of optimizing rate map spatial tuning, optimized how well the firing rate map predicted the BVC spiking. The second analysis also allowed us to ask whether that encoding is focused on a particular temporal horizon or whether the encoding captures behavior at multiple scales. To this end, for a given recording, we asked “How much time‐integration of the behavioral state is the observed spiking most consistent with?” We observed a wide spectrum of time‐constants of integration across cells, indicating that BVCs form a multiscale encoding of future states. The distribution of both offsets and integration rates observed across BVCs did not differ significantly from other, non‐BVC, subiculum neurons. Taken together, these findings indicate that BVCs, along with other subiculum neurons, form a multi‐scale encoding of future states.

## 1 Introduction

Cellular and oscillatory activity in the hippocampal formation exhibits robust correlations with navigational states, including position (O'Keefe and Dostrovsky 1971; Ekstrom et al. 2003; Ulanovsky and Moss 2007; Hafting et al. 2005), head direction (Taube et al. 1990), boundary proximity/direction (Lever et al. 2009; Solstad et al. 2008; Alexander et al. 2020), and locomotion, including running speed (Sargolini et al. 2006; Caplan et al. 2003; Wells et al. 2013). These correlations are typically derived by comparing the neural variable in question to the subject's instantaneous state, such as the firing rate of a place cell to the instantaneous position of the rat locomoting in an open field. Intriguingly, however, such neural correlations are often enhanced when spiking activity, for example, is aligned not with the animal's instantaneous state, but rather with its time‐shifted or time‐integrated state (Muller and Kubie 1989; Blair and Sharp 1995; Dannenberg et al. 2019; Bright et al. 2020; Chaudhuri‐Vayalambrone et al. 2023).

For example, the spatial tuning of CA1 neurons is more precise if aligned with the animal's position around 120 ms after the spike occurred, instead of its position at the time of the spike (Muller and Kubie 1989; Chaudhuri‐Vayalambrone et al. 2023). Similarly, spatial tuning in the subiculum (Sharp 1999) and in grid cells of the medial entorhinal cortex (Chaudhuri‐Vayalambrone et al. 2023) is enhanced by aligning spikes to future positions. These observations are consistent with the idea that the entorhinal‐hippocampal system is generating predictions about future states (Stachenfeld et al. 2017; Momennejad 2020; Geerts et al. 2023). However, not all coding in the entorhinal‐hippocampal system appears to be prospective. For instance, entorhinal speed cells, though obviously linked to locomotion, provide a retrospective record, encoding the historical running speed of the animal (Dannenberg et al. 2019). Furthermore, cells in the lateral entorhinal cortex also track the recent past, coding elapsed time since salient event boundaries (Bright et al. 2020; Tsao et al. 2018). Accordingly, it is not straightforward to suggest that entorhinal‐hippocampal codes are simply predictive.

Beyond the prospective‐retrospective dimension, a further important dimension of functional interest concerns multiple timescales. For instance, considering prospective coding, entorhinal grid cells encode the rat's future position with temporal offsets proportional to their spatial scale, thereby providing a multiscale representation of the animal's trajectory (Chaudhuri‐Vayalambrone et al. 2023). Considering retrospective coding: Speed cells encode the historical running speed of the animal at widely differing timescales ranging from hundreds of milliseconds to hundreds of seconds, depending on the neuron (Dannenberg et al. 2019); and lateral entorhinal cells encode the amount of time that has passed since critical event boundaries at multiple timescales (Bright et al. 2020; Tsao et al. 2018).

Theoretical modeling shows that multiscale encoding provides a rich basis upon which to form memories and predict future states (Howard et al. 2014; Shankar et al. 2016). A key prediction of that modeling is the presence of a spectrum of encoded timescales. Such a spectrum enables encoding of a memory timeline of *what happened when* and forming an estimate of the timeline of the future. The multiscale encodings observed in entorhinal neuronal activity (e.g., Tsao et al. 2018; Dannenberg et al. 2019; Bright et al. 2020; Chaudhuri‐Vayalambrone et al. 2023) exemplify functional types of spectral encoding. Importantly, in this theory, the type of information encoded determines the types of inferences obtainable. For example, multiscale encoding of time since event boundaries supports retrospective reconstruction of a timeline.

Boundary vector cells (BVCs) are spatially tuned neurons found in the subiculum that exhibit increased firing in the presence of environmental boundaries (e.g., wall or drop edge) or sufficiently large objects at specific allocentric distances and directions (Lever et al. 2009; Stewart et al. 2014; Poulter et al. 2021). The existence of such cells was first predicted by computational models of inputs to place cells (O'Keefe and Burgess 1996; Hartley et al. 2000; Lever, Burgess, et al. 2002) seeking to explain how place cell firing fields are shaped by environmental geometry (O'Keefe and Burgess 1996; Lever, Wills, et al. 2002). BVCs show some plasticity: for example, their firing fields can be influenced by the (e.g., rectangular) geometry of the environment (Muessig et al. 2024); a subtype of BVCs, called vector trace cells, also exhibit *memory* for the locations of boundaries and objects (Poulter et al. 2021). See (Wang and Bicanski 2026) for a computational model of how vector trace cells may emerge. Generally, less is known about subicular BVCs than about other cell types in the entorhinal‐hippocampal system, see (Poulter et al. 2018; Bicanski and Burgess 2018, 2020; Wang and Bicanski 2026; Lever et al. 2026) for reviews and discussion.

Whether subicular BVCs encode boundary vectors retrospectively or prospectively, and whether this code operates over a range of timescales, are open questions. To address these questions, we first applied rate‐map methods, like those used to reveal multiscale temporal coding in grid cells (Chaudhuri‐Vayalambrone et al. 2023), to estimate the temporal offset between BVC spiking and position for each cell that maximizes spatial tuning in the rate‐map. In a complementary approach, we modeled the spiking of each BVC as a function of a time‐shifted and/or time‐integrated representation of the rat's position. Model variants with a free parameter for time‐shift test whether BVC spiking is more predictable when the position was shifted forward or backwards in time and, if so, provide insight into the offsets that maximize that predictability. Model variants with a free parameter enabling time‐integration of position test whether BVC spiking is most consistent with a narrow (e.g., instantaneous) estimate of the rat's position or whether it tracks a position estimate obtained by averaging over a temporal window with some width. If the latter, these models also provide insight into the nature and scale of that temporal horizon. Our results, across analysis approaches, demonstrate that BVC spiking is best explained as time‐integrated estimates of *future* position but that, across cells, there is a spectrum of temporal offsets and temporal scales encoded.

## 2 Methods

New analyses were performed on recordings obtained by and first described by Lever et al. (2009). Subjects, surgery, and data collection are paraphrased here for ease of reference.

### 2.1 Subjects and Surgery

Data were obtained from six male Lister Hooded rats, weighing 315–390 g at the time of surgery. Rats were maintained on a 12:12 h light:dark schedule (with lights off at 15:00). Food deprivation was maintained such that subjects weighed 85%–90% of free feeding weight. Recordings were made from the dorsal subiculum. Details of the implantation and confirmation of electrode placement were described previously (Cacucci et al. 2004; Lever et al. 2009). Briefly, a bundle of four HM‐L coated platinum‐iridium wire tetrodes mounted to a microdrive was chronically implanted dorsal to the subiculum under deep anesthesia. Tetrodes were lowered to isolate cells after surgery.

### 2.2 Electrophysiology, Location and Head Orientation

Tetrodes were lowered toward the subiculum region and then left to stabilize before the start of the recording. The head stage was connected to a pre‐amplifier by 3‐m lightweight wire. The outputs of the pre‐amplifier passed through a switching matrix, and then to the filters and amplifiers of the recording system. Each channel was continuously monitored at a sampling rate of 50 kHz and action potentials were stored as 50 points per channel whenever the signal from any of the 4 channels of a tetrode exceeded a given threshold. EEG signals were amplified 10–20 K, band‐pass filtered at 0.34–125 Hz and sampled at 250 Hz (See details in Lever et al. 2009).

Head position and orientation were tracked using an overhead video camera and tracking hardware/software by tracking the position of two arrays of head‐mounted small, infrared LEDs, one array brighter and more widely projecting than the other. Cluster cutting to isolate single units was performed manually using custom made software (TINT, Axona, UK). (See details in Lever et al. 2009). For all analyses, the complete record of position was used. Though there is precedent for exclusion of data from epochs of low or no velocity, in the context of time shifting and time integrating behavior, this practice would have introduced undue complexity to the methods and reduced overall transparency.

### 2.3 Experimental Design and Training

Recordings were collected as rats foraged for cereal in multiple distinct open field arenas. Rats were transferred to the arenas in a consistent fashion to maintain orientation. At the end of each trial, the rat was removed from the recording environment and placed back on the holding platform until the next trial. The length of the trial was proportional to the size of the arena and was in the range 10–16 min. The inter‐trial interval was 20 min. The arena specifics are described by Lever et al. (2009). In all recordings, an external white cue card (102 cm high, 77 cm wide) provided directional constancy.

### 2.4 Rate Map Creation

Firing rate maps were constructed from 2.6 × 2.6 cm binned data, smoothed using a Gaussian smoothing kernel with a standard deviation of 5.2 cm. Spike count was divided by occupancy time to obtain a firing rate per bin. To display firing rate maps in figures, the rate maps were auto‐scaled false color maps, each color representing a 15% band of peak firing rate, from dark blue (0%–15%) to red (85%–100%). Peak rate (after smoothing) is shown next to each rate map.

### 2.5 Rate Map Tuning Based Approaches Estimating Time‐Shift

Following previously used approaches for estimating the temporal lag of spatial coding, we examined results from spatial information (s.i.) (Skaggs et al. 1993) and zero‐lag spatial autocorrelation (ZLAC) (Chaudhuri‐Vayalambrone et al. 2023) approaches with variable temporal offsets between the tracking and spiking. S.i. is computed as,

(1) $$s.i.=\sum_{n}p_{n}\;\frac{r_{n}}{\bar{r}}\;\log_{2}\;\left(\frac{r_{n}}{\bar{r}}\right)$$

where $p_{n}$ is the probability of occupying bin n, $r_{n}$ is the smoothed mean firing rate at bin n, and $\bar{r}$ is the mean overall firing rate. This form of s.i. has the units of bits per spike. Note, however, that because the optimization was performed for each recording separately, preserving the recording duration and total spikes, it would not have changed the qualitative results had it been performed with the bits per second variant. ZLAC in this context is equivalent to the mean squared value of each rate map, computed as,

(2) $$\text{ZLAC}=\frac{1}{N}\sum_{n}r_{n}^{2}$$

where $r_{n}$ is the smoothed mean firing rate at bin n (i.e., the nth bin of the rate map). The maximum s.i. and ZLAC scores are obtained when spikes are least uniformly distributed over spatial bins.

**FIGURE 1:**
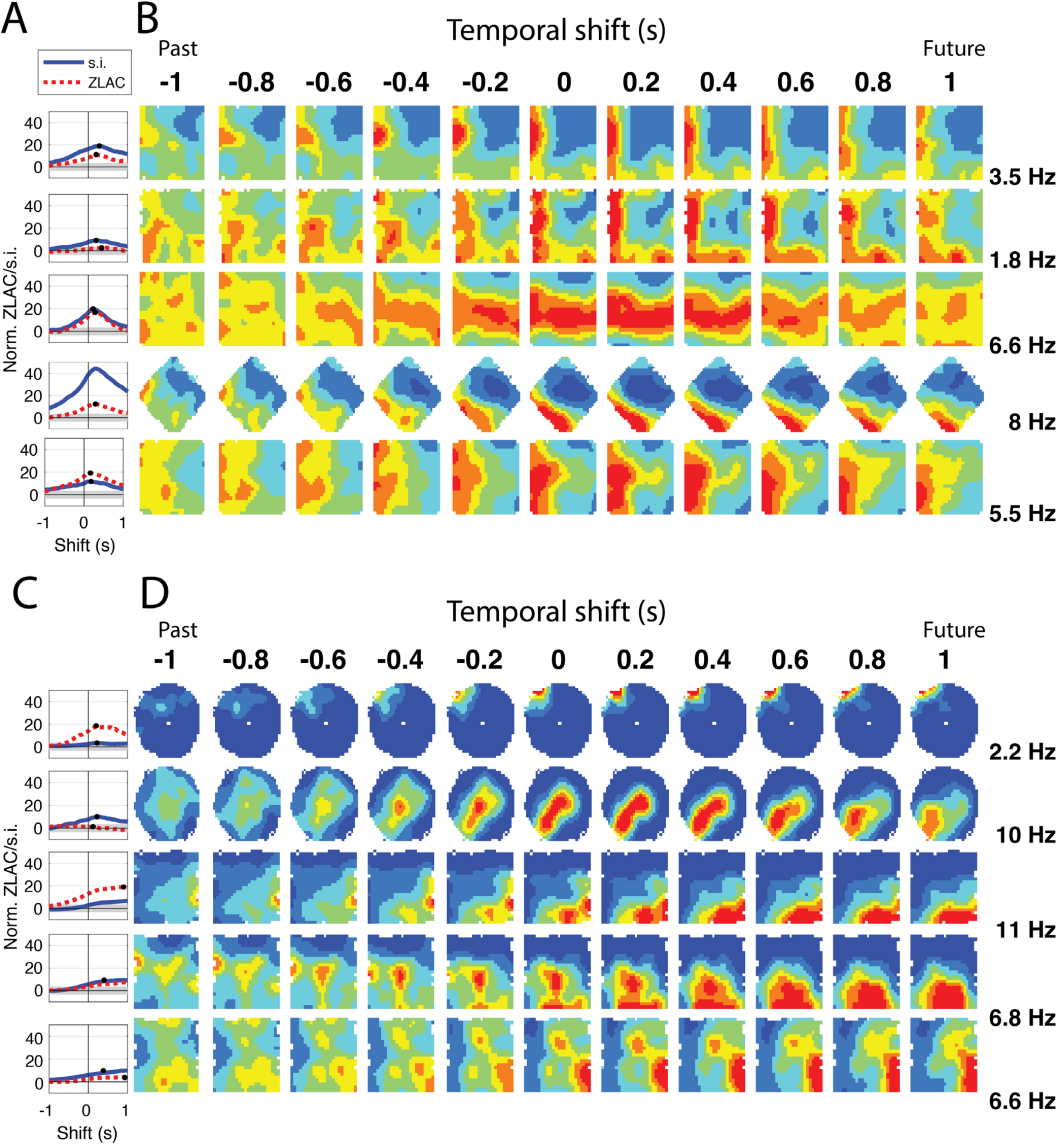
Time shifting spikes relative to rat position improves spatial tuning. (A) Normalized (z‐scored) spatial information (s.i.; solid line) and normalized zero‐lag auto‐correlation(ZLAC; dashed line) for five representative BVCs are shown, plotted as a function of the temporal shift between the spiking and spatial position. Black dots mark the lag that resulted in the peak value for each cell. (B) Spatial firing ratemaps for a subset of the points shown for each of the aligned curves in A. A fixed color map is used across all ratemaps scaled to the peak firing rate observed at any shift, indicated at the right of each row. (C, D) same as A‐B but for representative non‐BVCs recorded in the subiculum.

In both approaches, each recording for each cell was analyzed separately. For each, we shifted the timing of the spiking relative to the tracking one video frame (20 ms) at a time for up to 2 s into the past and into the future. For each step, we created a rate map and then computed the s.i. and ZLAC scores. Over all steps, a curve of scores was constructed. These curves were then smoothed with a boxcar kernel with a width of 6 (120 ms). Representative rate maps and curves are shown in Figure 1. Significance of s.i. and ZLAC at each lag was established by performing 500 pseudo‐random rotations between the tracking and spiking to establish the null distributions of s.i. and ZLAC for each recording. The rotations were constrained to shift the relative alignment of the data types by at least 5% of the recording length (30 s in the case of the 10 min recordings). The ‘rotation’ here is the wrapping of the misaligned data from one end of the recording around to the other. The permuted ZLAC and s.i. scores were used to compute a z‐score type normalization of the empirical ZLAC and s.i. values, respectively. Normalized values were considered to reflect significant spatial tuning if they were larger than 3.4808, reflecting p<0.00025 (the Bonferroni corrected p<0.05 over 200 comparisons). From each of the normalized curves with significant values, we identified the local maxima closest to zero as the ‘peak shift’ for that recording. The peak shifts across different recordings of the same cell were then averaged to establish a per‐cell‐estimate so as to perform statistics across cells instead of recordings.

### 2.6 Maximum Likelihood Estimation of Spatio‐Temporal Tuning

A concern with the approach described above is that it maximizes how non‐uniform the rate map is without consideration for whether that resulting rate map accurately captures the firing statistics of the neuron. To address this, we performed a complementary analysis approach that modeled the spiking of each cell as a function of a time‐shifted and/or time‐integrated representation of the rat's position. In this approach, temporal tuning parameters were selected so that the observed firing in a recording was maximally likely given knowledge of the rat's trajectory. Model variants with a free parameter for time‐shift test whether BVC spiking is more predictable when the position was shifted forward or backwards in time and, if so, provide insight into whether the neuron has prospective or retrospective coding. Model variants with a free parameter enabling time‐integration of position test whether BVC spiking is most consistent with a narrow (e.g., instantaneous) estimate of the rat's position or whether it tracks a position estimate obtained by averaging over a temporal window with some width. If the latter, these models also provide insight into the nature and scale of that temporal horizon.

In this approach, the quality of a model using a given set of parameters was assessed as follows:

1. Compute a surrogate of the time‐varying position of the rat as follows:
(3) $${\tilde{x}}_{t}=f\left(t;\tau ,\kappa \right)$$
  where τ is the magnitude and direction (future or past) of a time‐shift and κ controls the width of a time integration window. How this was done specifically differed over analyses and is unpacked for each specific analysis below.
2. For a given set of surrogate ${\tilde{x}}_{t}$ values, build a rate map as per the methods described above.
3. Predict the probability of observing a spike in each time‐bin: Using the rate map as a look‐up table of the expected firing rate based on position, identify the current expected firing rate given the current surrogate position, ${\tilde{x}}_{t}$, and then convert that firing rates into the probability of observing a spike using the Poisson probability mass function. This is achieved via the following,

(4) $${\hat{p}}_{\text{spike}}=1-e^{-r\left({\tilde{x}}_{t}\right)\cdot \mathrm{dt}}$$

where $r\left(\tilde{x}\right)$ is the rate map value at surrogate position $\tilde{x}$ and dt is the bin‐width used in discretizing the predictions, set to 0.02 s (one frame of the tracking).

4 Compute the total negative log‐likelihood (NLL) for the entire time series. Because the model treats each time bin as an independent Bernoulli trial, the total NLL is the sum of the NLLs for each individual bin. This is calculated as follows:

(5) $$\begin{array}{c} L\left(y,{\hat{p}}_{\text{spike}}\right)=-\sum_{t}\left[y_{t}\log\left({\hat{p}}_{\text{spike},t}\right)+\left(1-y_{t}\right)\log\left(1-{\hat{p}}_{\text{spike},t}\right)\right] \end{array}$$

where the sum is over all time bins t in the recording, y is the vector of observed binary spike data (where $y_{t}\in \left\{0,1\right\}$), and ${\hat{p}}_{\text{spike}}$ is the corresponding vector of the model's predicted spike probabilities. This calculation yields a single scalar value that quantifies the goodness‐of‐fit for a given set of parameters, τ and κ. Lower $L\left(y,{\hat{p}}_{\text{spike}}\right)$ values indicate better predictions of the observed spike train.

This approach enables testing of whether a given parameter significantly improves how well the rate map obtained with given values of τ and κ predicts the observed spiking. Broadly, this is done by testing whether $L\left(y,{\hat{p}}_{\text{spike}}\right)$ is significantly better when a parameter is treated as a free‐parameter (i.e., one that can be optimized via a gradient descent algorithm) instead of being fixed to a default value. Details of statistical testing are described below.

#### 2.6.1 Time‐Shift

The time‐shift parameter, τ, temporarily offsets the spiking with respect to the behavioral tracking. A value of 0 corresponds to how standard rate maps, with no temporal shift, are constructed. Positive τ values align spiking with future positions (i.e., predict spiking from where the rat will be). Negative values align spiking to past positions (predict spiking from where that rat was). We tested whether optimizing τ generated rate maps that reliably predicted spiking better than those generated with no time‐shift, and if so, whether that offset was reliably offset toward the future or the past as described in Statistical Testing.

#### 2.6.2 Time‐Integration

Time‐integration estimates each position based on a time‐integrated version of position over a recent window with width proportional to κ. The form of the time‐integration depends on the shape of the kernel used.

We compared three kernel shapes: boxcar, Gaussian, and exponential. Each was compared to a baseline model with no integration.

The generalized form for the smoothed estimate at time t is:

(6) $$\tilde{x}\left(t;\tau ,\kappa \right)\;=\;\frac{\sum_{s\in W\left(t,\tau \right)}K_{\kappa }\;\left(s-\left(t+\tau \right)\right)\;x\left(s\right)}{\sum_{s\in W\left(t,\tau \right)}K_{\kappa }\;\left(s-\left(t+\tau \right)\right)}$$

where $K_{\kappa }\left(x\right)$ is a given kernel function with width controlled by the parameter κ. To prevent information leakage between training and test folds during cross‐validation, the integration window $W\left(t,\tau \right)$ was bounded to include only samples within 15 s of the shifted time point t+τ. For all models, if a kernel's effective window extended beyond this bound, it was truncated to $W\left(t,\tau \right)$ and renormalized by the denominator in Equation (6).

##### 2.6.2.1 Boxcar (Uniform)

This model implements the simpler hypothesis that a neuron is equally sensitive to all behavioral states within a defined window. This corresponds to a uniform kernel applied retrospectively from the time point t+τ:

(7) $${\tilde{x}}_{\text{Boxcar}}\left(t;\tau ,\kappa \right)\;=\;\frac{1}{\kappa }\sum_{k=0}^{\kappa -1}x\;\left(t+\tau -k\right)$$

where the window width, κ, is the number of time bins included in the average. During optimization, κ was bounded to 15,000 ms.

##### 2.6.2.2 Gaussian

This model tests the hypothesis that neural activity is maximally sensitive to behavior at latency τ, with decreasing sensitivity for times further from this point. This is implemented with a Gaussian kernel:

(8) $$K_{\kappa }\left(u\right)\;=\;\exp\left(-\frac{u^{2}}{2\sigma^{2}}\right),\;\text{where}\;\sigma \equiv \kappa$$

This symmetric kernel integrates states both before and after the central time point t+τ. Such a formulation is consistent with theoretical work on scale‐invariant representations of time that can be adapted to encode space O'Keefe and Burgess (1996). During optimization, the standard deviation σ was bounded to $\left[10,100000\right]$ ms.

##### 2.6.2.3 Exponential

To capture potentially asymmetric temporal sensitivity (i.e., a retrospective or prospective bias), we used a one‐sided exponentially decaying kernel. The model's direction (past vs. future) and decay rate were governed by a single fitted parameter, ω, which was bounded to $\left[-12,12\right]$. This parameterization avoids the numerical instability that can arise when fitting the direction and decay rate separately. Specifically, we mapped ω to a decay constant κ and a direction d:

(9) $$\kappa \;=\;\frac{1}{|\omega |+\varepsilon },\;d\;=\mathrm{sign}\left(\omega \right)\in \left\{-1,+1\right\}$$

where a small constant ε ensures a minimal decay rate. The estimated behavioral state is then given by:

(10) $${\tilde{x}}_{\mathrm{Exp}}\left(t;\tau ,\omega \right)\;=\;\frac{\sum_{k=0}^{K_{\max}}\lambda^{k}\;x\;\left(t+\tau +d\;k\right)}{\sum_{k=0}^{K_{\max}}\lambda^{k}},\;\text{where}\;\lambda =\exp\left(-\frac{\Delta t}{\kappa }\right)$$

here k indexes bins, $K_{\max}=\left\lfloor \frac{15000\;\mathrm{ms}}{\Delta t}\right\rfloor$ to set the number of bins to sum over to enforce the 15,000 ms bound, d=+1 applies the kernel to future samples (prospective model) and d=−1 applies it to past samples (retrospective model).

#### 2.6.3 Parameter Optimization

To optimize the free parameters for each model, we used a particle swarm gradient descent approach. Specifically, we used the Matlab function particleswarm.m with SwarmSize set to 50 and HybridFcn set to fmincon, which implements a local gradient‐based search after the initial swarm exploration. To ensure robust fitting, a random restart procedure was used wherein the particle swarm was reinitialized iteratively until five consecutive restarts failed to produce an improved fit. The parameter set that yielded the lowest final negative log‐likelihood ($ℒ\left(y,{\hat{p}}_{\text{spike}}\right)$) on the training data was kept for subsequent analysis.

### 2.7 Cross‐Validation

To test whether optimized parameters were generalizable to data not used in their estimation, and thereby support the critical evaluation of the goodness of the model fits, we used a K‐fold cross‐validation approach with K=10. That is, recordings were split into 10 consecutive segments along the time variable. On each of 10 separate runs, a different segment was omitted from the fitting process and the remaining K−1 data segments were used for parameter estimation. The omitted segment was used as the validation dataset, used to assess the quality of the resulting fit.

Time‐shifting and time‐integrating procedures can blur the boundaries between the fitting and validation segments and introduce segments at the start or end of the recording without aligned behavior and spiking. To address this, buffers were cut from the start and end of the recording and around the validation dataset. Critically, a buffer of fixed size was used across all parameterizations. This was so that the number of omitted data points was constant across parameterizations and the likelihood score could not be affected by varying numbers of samples across parameter values.

### 2.8 Parameter Aggregation

Individual parameters were separately estimated for each cell dozens of times as a result of each of the recordings of that cell being subjected to a 10‐fold cross validation testing protocol. After establishing that the parameter estimate stability was good to excellent (see section below on *Parameter reliability with ICC*), a single estimate of each parameter was obtained by taking the median value over all the estimates to enable analysis of parameter distributions across cells.

### 2.9 Statistical Testing

We employed different statistical approaches across analyses as follows: We used the non‐parametric Wilcoxon signed‐rank test to test for significant improvements in the rate map based analyses. For the maximum‐likelihood (ML) models, we used the Bayesian Information Criterion (BIC) for within‐dataset model selection. The BIC score for a model improves (reduces) in two circumstances: (1) the model is more accurate at predicting the data, and (2) the model uses fewer free parameters. Thus, it helps select for the models that are most parsimonious yet account for the data well. Population‐level effects were assessed with a Linear Mixed‐Effects (LME) model. Parameter estimate reliability was assessed with intra‐class correlations (ICC). Each is described here.

#### 2.9.1 Rate Map–Based Analysis

The Wilcoxon signed‐rank test (reported as a $W_{\mathrm{sr}}$ statistic) was used to compare distributions to zero or to perform paired tests. The Wilcoxon rank‐sum test (reported as a $W_{\mathrm{rs}}$ statistic) was used for testing between independent samples.

#### 2.9.2 Maximum‐Likelihood Model Comparison

With the goal of selecting a method of building rate maps that were maximally predictive of spiking that was not overly complicated, we compared different models on the basis of their Bayesian Information Criterion (BIC) scores. The BIC score for a model improves (reduces) in two circumstances: (1) the model is more accurate at predicting the data, and (2) the model uses fewer free parameters. It is computed as follows:

(11) $$\mathrm{BIC}=k\ln\left(n\right)-2\ln\left({\hat{L}}_{\text{test}}\right)$$

where k is the number of free parameters, n is the number of time bins, and ${\hat{L}}_{\text{test}}$ is the likelihood of the spiking observed in the test dataset under the optimized parameters. The number of free parameters was either zero in the case of the standard rate map, one models with either a time shift (τ) or a time‐integration window (κ), and models with both terms added two.

To assess whether one parameter or the other reliably improved the BIC, a two‐factor repeated‐measures analysis of variance was conducted to examine the effects of adding a shift and varying the time‐integration approach. Time shift (2 levels: none vs. optimized) and kernel (4 levels: none, uniform, Gaussian, exponential) were included as fixed factors, and their interaction was tested to determine whether the effect of temporal shifting depended on kernel type. Because multiple estimates were obtained from the same neuron, neuron identity was included as a random intercept in a linear mixed‐effects framework to account for the non‐independence of repeated estimates within cells. This specification models cross‐validation and trial‐level observations while performing statistical inference at the level of independent neurons.

#### 2.9.3 Population‐Level Inference With LME

To ask whether particular temporal components (time shift and/or the choice of integration kernel) reliably improved predictive performance across a cell population (BVCs or non‐BVCs), we fit a Linear Mixed‐Effects (LME) model to the cross‐validated test‐set negative log‐likelihood (NLL), reducing sensitivity to overfitting:

(12) $${\mathrm{nll}}_{\text{test}}∼T_{\text{shift}}\times T_{\text{kern}}+\left({\bar{r}}_{\mathrm{spk}}\;|\;\text{cellID}\right)$$

where $T_{\text{shift}}\in \left\{0,1\right\}$ indicates whether a free time‐shift parameter was included, and $T_{\text{kern}}\in \left\{\text{none},\text{boxcar},\text{exponential},\text{normal}\right\}$ indexes the temporal‐integration kernel, ${\bar{r}}_{\mathrm{spk}}$ is the average spike rate for each recording, and cellID indicates which cell each recording is from. In practice, this asks whether our ability to predict the spiking of a cell for a withheld portion of data (${\mathrm{nll}}_{\text{test}}$) varied significantly when we optimized $T_{\text{shift}}$ and/or used particular temporal‐integration kernel. Performing this LME fits beta weights for each term (i.e., how much ${\mathrm{nll}}_{\text{test}}$ changes as a function of each). Whether the beta weights are significantly different from zero indicates whether those terms had a significant impact on ${\mathrm{nll}}_{\text{test}}$. Negative beta weights indicate that a term improved the fit. The $\left({\bar{r}}_{\mathrm{spk}}|\;\text{cellID}\right)$ term defines a spike rate and cellID as random effects. As a result, statistical inference is performed at the level of cells, rather than trials or fits, while still leveraging within‐cell information to improve estimation precision.

The specific equation used to perform the LME analysis is itself another model and can be more or less complex. In the form we use here, the model included a random slope for the mean firing rate of the cell, ${\bar{r}}_{\mathrm{spk}}$, to factor out variance in ${\mathrm{nll}}_{\text{test}}$ related to firing rate before assessing the impact of each of the other terms. This is more complex than the simplest possible model,

(13) $${\mathrm{nll}}_{\text{test}}∼T_{\text{shift}}\times T_{\text{kern}}+\left(1|\text{cellID}\right)$$

We used it nonetheless because the BIC was lower (BIC $=2.69\times 10^{5}$ vs. $2.94\times 10^{5}$), indicating that the cost of the extra term was outweighed by the improvement predicting the ${\mathrm{nll}}_{\text{test}}$. The qualitative pattern of results reported here did not depend on including mean spike rate as a random effect.

#### 2.9.4 Parameter Reliability With ICC

To establish whether parameter fits were reliable (i.e., consistent) across cross‐validation folds and recordings of the same cell, we used intra‐class correlation (ICC) (Shrout and Fleiss 1979; Koo and Li 2016). We used the form to test for one‐way random effects, absolute agreement, multiple raters/measurements (i.e., ICC(1,k) approach). To test for stability at the broadest level, all parameter estimates for a given cell (10 k‐fold fits for each of the separate recordings of the same cell) were treated as independent raters. To test for stability across recordings of the same cell, only one of the K‐fold estimates were used from each of the recordings (the first) for each cell. Per convention, values less than 0.5 indicate poor reliability, between 0.5 and 0.75 indicate moderate reliability, between 0.75 and 0.9 indicate good reliability, and greater than 0.9 are indicative of excellent reliability (Koo and Li 2016). We found the parameter estimates had good to excellent reliability (i.e., broadly consistent) across the k‐fold cross‐validation runs and between separate recordings (see Table 1).

**TABLE 1:** Intra‐class correlations (ICC) for the time lag and time integration parameter estimates across model types to assess fit reliability.

ICC values	Integration type	None	Boxcar	Normal	Exponential
	Parameter	wo/w shift	wo/w shift	wo/w shift	wo/w shift
All estimates	Time lag	ø/0.97	ø/0.96	ø/0.87	ø/0.96
	Time int.	ø/ø	0.99/0.99	0.97/0.95	0.97/0.96
Across recs.	Time lag	ø/0.82	ø/0.88	ø/0.92	ø/0.91
	Time int.	ø/ø	0.93/0.93	0.87/0.83	0.87/0.87

*Note:* Model types are defined by the kernel type listed in the top row and whether they were without (wo) or with a time shift (w shift). Reliability across k‐folds is shown in rows labeled with ‘All estimates.’ Reliability across recordings was computed by running ICC on a single cross‐validation estimate across recordings for each cell and is shown in rows labeled with ‘Across recs.’ A ø is shown for models wherein the specific parameter was not estimated.

Abbreviations: DF, degrees of freedom; ICC, intra‐class correlation; int, integration; recs, recordings.

## 3 Results

### 3.1 BVC Spatial Tuning Is Greater for Future Positions

Our first approach to assessing if BVCs encode boundary vectors retrospectively or prospectively was to ask whether, for each, the spatial tuning observable in the rate map was more structured when the spiking was temporally offset in one direction or the other relative to the behavior. This was repeated at varying magnitudes of offset. Figure 1 shows representative examples of how the rate maps changed as a function of temporal offset (note that only a subset of the offsets tested are illustrated here). To quantify changes in spatial tuning across offsets, we used spatial information (s.i.) and the zero‐lag auto‐correlation (ZLAC), metrics that increase when spiking is less uniform. Specifically, we used the offset that yielded the maximum score to assess whether each cell encoded boundary vectors prospectively or retrospectively.

**FIGURE 2:**
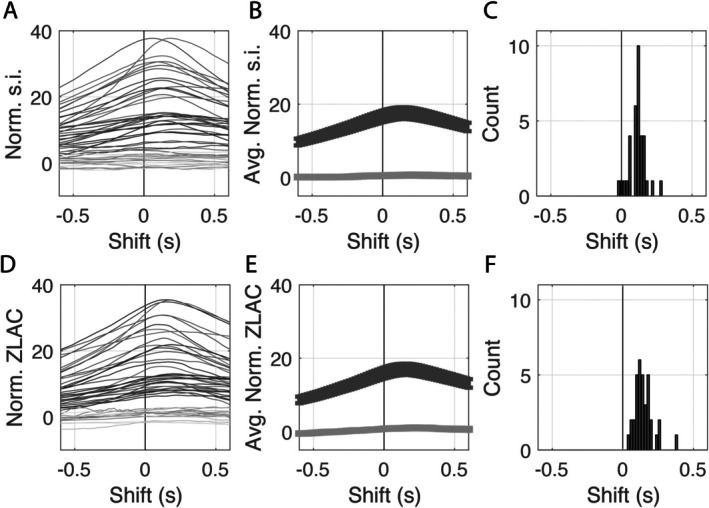
Temporal offsets that align spiking with future behavior yield rate maps with peak normalized spatial information (s.i.) and zero‐lag autocorrelation (ZLAC). (A) Mean normalized s.i. as a function of temporal lags plotted separately for all recordings from each BVC with at least one significant normalized s.i. (black) and recordings without significant normalized s.i. (gray). Positive aligns spikes with future positions. (B) Mean standardized s.i. for recordings with significant (black) and non‐significant (gray) spatial tuning. Confidence intervals indicate standard error. (C) Histogram of the temporal offsets that resulted in the peak normalized s.i. scores. (D–F) Same as A–C but for ZLAC instead of s.i.

The mean offsets over recordings for each cell that generated peak s.i. values were reliably positive across the 35 BVCs (33 of 35 were positive, median [CI] = 120 ms [110,130], Wilcoxon sign‐rank $W_{\mathrm{sr}}=594,Z=5.07,p<0.001$). Likewise for ZLAC, the offsets that generated the maximum values were also reliably positive (35 of 35 were positive, median [CI] = 150 ms [120,170], $W_{\mathrm{sr}}=630,Z=5.16,p<0.001$). Thus, both s.i. and ZLAC had peaks at an offset of about one theta cycle into the future. These results are shown in Figure 2. The fact that rate maps had the largest s.i. and ZLAC at positive offsets indicates that BVC spiking is most informative about future positions.

**FIGURE 3:**
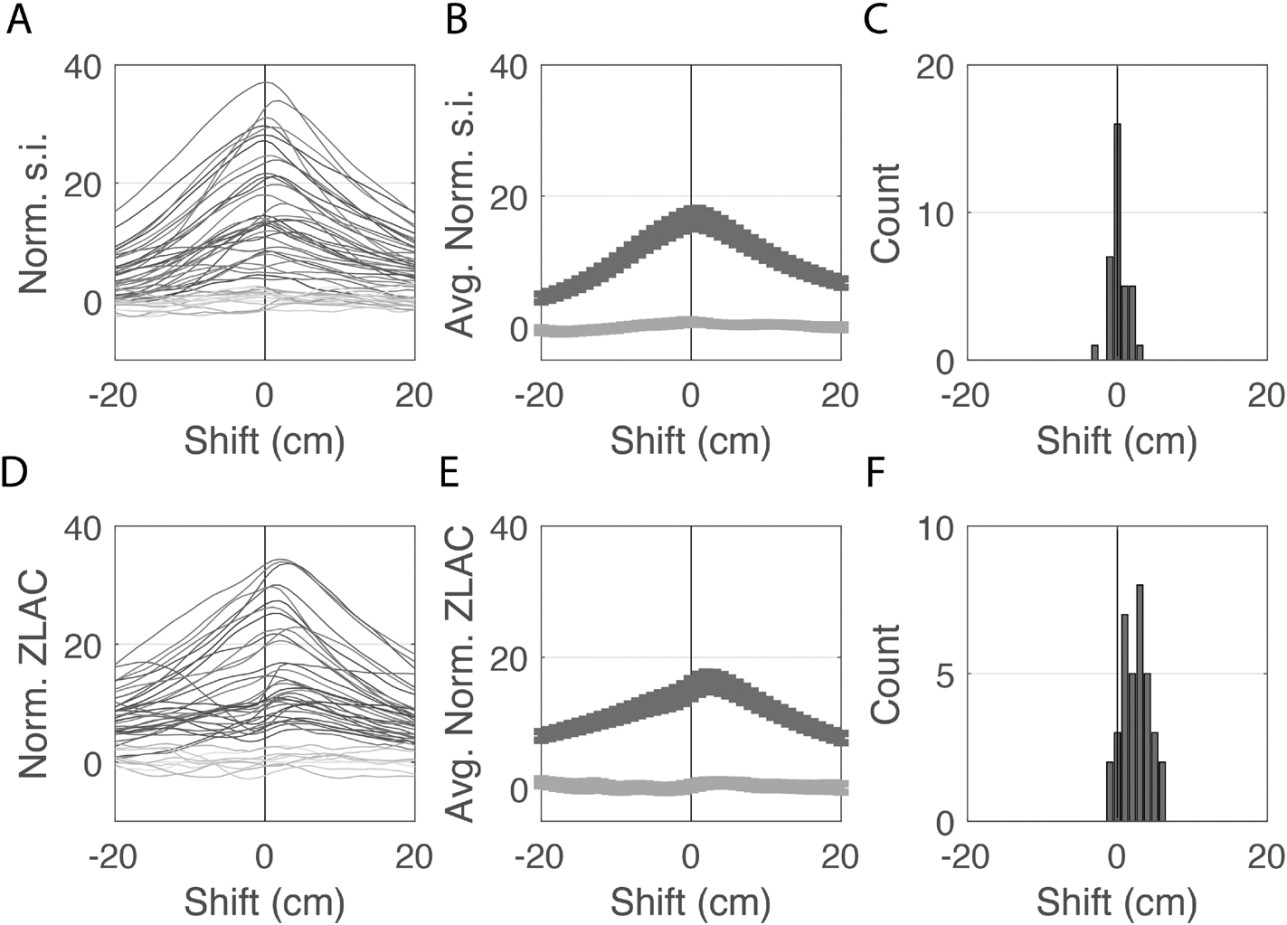
Spatial offsets that yielded peak normalized s.i. and ZLAC aligned BVC spiking with future positions. (A) normalized s.i. as a function of spatial lags plotted separately for all recordings from each BVC with at least one significant normalized s.i. (black) and recordings without significant normalized s.i. (gray). Positive aligns spikes with future positions. (B) Mean standardized s.i. for recordings with significant (black) and non‐significant (gray) spatial tuning. Confidence intervals indicate standard error. (C) Histogram of the spatial offsets that resulted in the peak normalized s.i. scores. (D–F) Same as A–C but for ZLAC instead of s.i.

The analyses above assume that offsets between BVC spiking and position are temporal (i.e., a fixed time shift) but the offset could, alternatively, be spatial (e.g., displaced by the distance between the head and the body). The key difference is that spatial shifts, but not temporal shifts, depend on running speed. The observed temporal offsets (120 to 150 ms) would, given the median running speed of 15.70 cm/s (95% CI = [14.87 16.85]), correspond to a spatial offset of 2.04 cm [1.93 2.19]. Testing what spatial shift, ranging from −20 to 20 cm in 0.5 cm increments, maximized s.i., we found the peak s.i. did not differ significantly from zero (12 of 35 were positive, $W_{\mathrm{sr}}=156,Z=1.43,p\;>0.05$). The median optimal shift was 0.0 cm (95% CI = [0.0 0.3]). ZLAC was largest when spiking was aligned with future positions (32 of 35 were positive, $W_{\mathrm{sr}}=555,Z=4.93,p<0.001$) albeit at larger offsets (median [CI] = 2.5 cm [2.0 3.5]). The results are shown in Figure 3.

Comparing the spatial tuning improvement magnitudes between the temporal versus spatial offsets, we found that the temporal offsets produced significantly larger improvements in s.i. (median difference [CI] = 0.92 bits [0.76 1.09], $W_{\mathrm{sr}}=560,Z=4.01,p<0.001$) and ZLAC (median difference [CI] = 0.55 [0.23 0.90], $W_{\mathrm{sr}}=442,Z=2.08,p<0.05$). These results indicate that the spatial offsets are not better than temporal offsets in terms of improving the apparent spatial tuning of the rate map.

**FIGURE 4:**
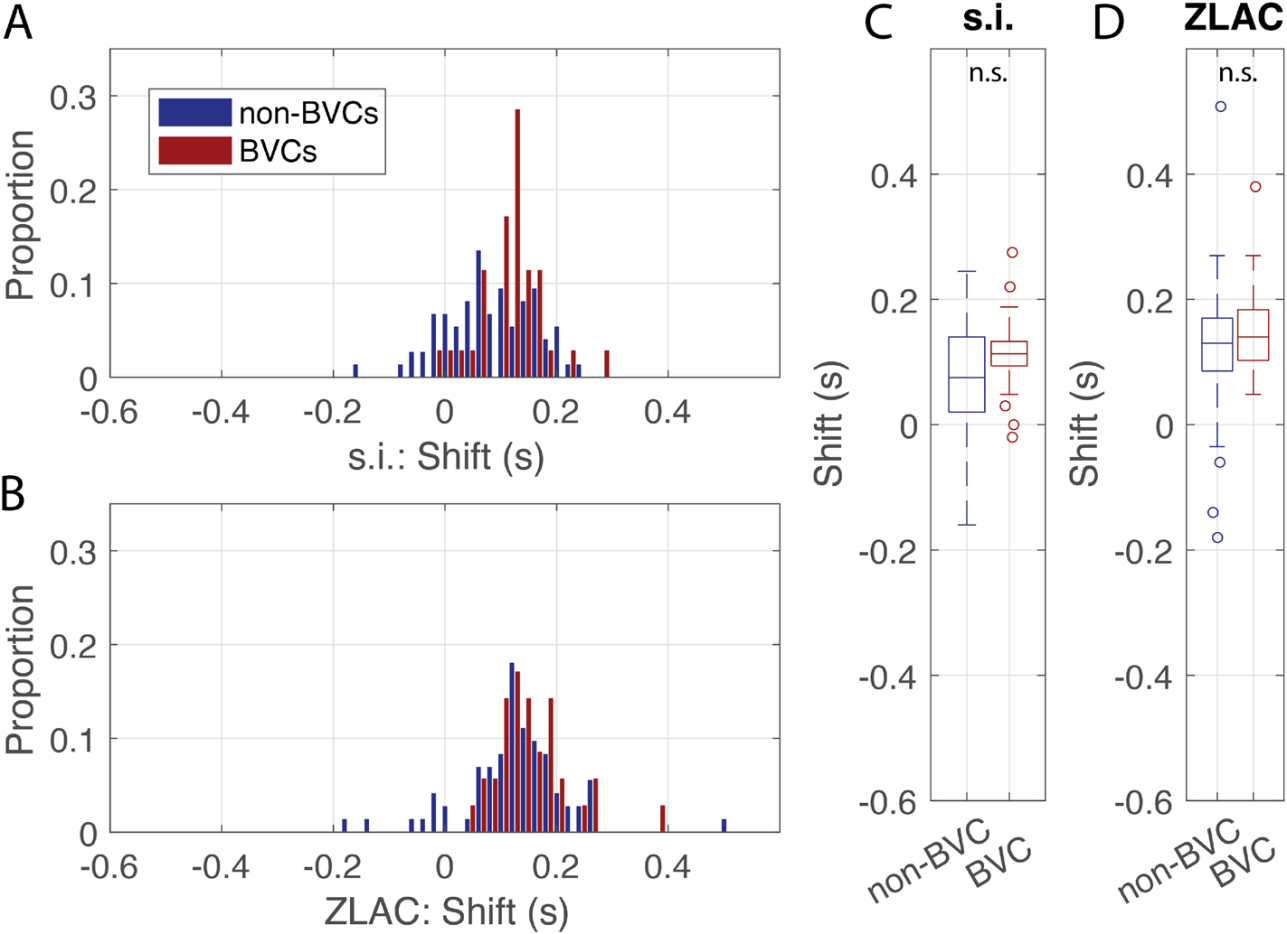
Spatial tuning in BVCs and non‐BVC ratemaps was maximized with similar temporal shifts. (A) Normalized histograms of shifts that yielded the maximum ratemap spatial information (s.i.) are shown for non‐BVCs (blue) and BVCs (red). (B) Same as A, but for shifts that yielded the maximum zero‐lag auto‐correlation (ZLAC). (C) Box‐and‐whisker plot comparisons of non‐BVCs BVCs in terms of the shifts that yielded the peak s.i. score. No significant difference was found. (D) Same as C, but for shifts that yielded the peak ZLAC score. No significant difference was found.

To gain perspective on the temporal tuning of these BVCs, we examined the tunings of non‐BVCs that were co‐localized with the BVCs analyzed above. Of the 78 unique non‐BVCs co‐located with BVCs, 74 (95%) had rate maps with significantly greater s.i. than chance for at least one temporal offset. The temporal offsets that generated the greatest s.i. scores for the non‐BVCs were, like the BVCs, reliably positive (60 of 74, 82%, were positive, median [95% c.i.] = 90 ms [60, 116], $W_{\mathrm{sr}}=2464,Z=6.13,p<0.001$). Using ZLAC to assess spatial tuning yielded a similar result. Of the 72 cells that had significant ZLAC scores at least one temporal offset (92%), the temporal offsets that generated the greatest ZLAC scores were reliably positive (63 of 72, 92% were positive, median [95% c.i.] = 130 ms [110140], $W_{\mathrm{sr}}=2442,Z=6.67,p<0.001$). Comparing the median optimal shifts from the BVCs and non‐BVCs, we found no significant differences for either s.i. ($W_{\mathrm{rs}}=3780,Z=-1.88,p=n.s.$) or ZLAC ($W_{\mathrm{rs}}=3657,Z=-1.53,p=n.s.$). See Figure 4 for a comparison. Thus, the BVCs share a similar magnitude bias toward future coding with other non‐BVC neurons of the subiculum, indicating coherent temporal coding across the area.

### 3.2 BVC Spiking Is Better Predicted by Future Position

The results above, showing that spatial tuning in rate maps increases when spiking is aligned with future positions, do not address whether those rate maps are more accurate descriptions of the spiking. Addressing this, we tested whether rate maps constructed with a temporal shift between the behavior and spiking were better able to predict neuronal spiking than the standard, non‐shifted rate maps.

For each recording, we estimated the optimal temporal shift and constructed a corresponding rate map using 90% of the data and then tested how well that rate map predicted spiking in the remaining data. Prediction accuracy was quantified using Bayesian Information Criterion (BIC), a metric which rewards prediction quality while punishing model complexity and, thereby, assesses whether the extra complexity is warranted given the change in prediction quality. See methods for full details. BIC was significantly improved (lowered) by building rate maps with an optimized temporal offset (*F*(3, 15,250) = 5.21; p = 0.02). See Table 2 for the full set of results. That is, when we used 90% of a recording to estimate the optimal temporal offset and then built a rate map from that data with that offset, our ability to predict the spiking in the remaining 10% was significantly better than if the rate map was constructed without a temporal offset even after adjusting for the complexity increase associated with adding the temporal offset.

**TABLE 2:** Linear mixed‐effects modeling results of how BVC spike prediction depended on temporal lag and integration approach.

	BIC	Effect	C.I.	dF	p
No lag, no int.	13,476		$\left[11483,15470\right]$	15,432	
+ time lag		−104.0	$\left[-192.5,-15.7\right]$	15,432	<0.05
+ boxcar int.		−845.5	$\left[-934.8,-756.1\right]$	15,432	<0.001
+ normal int.		−940.1	$\left[-1029.4,-850.8\right]$	15,432	<0.001
+ exp. int.		−812.0	$\left[-901.3,-722.6\right]$	15,432	<0.001
lag X boxcar int.		−90.5	$\left[-215.5,34.5\right]$	15,432	n.s.
lag X normal int.		−38.4	$\left[-163.4,86.5\right]$	15,432	n.s.
lag X exp. int.		−785.9	$\left[-910.8,-660.9\right]$	15,432	<0.001

*Note:* The BIC is the Bayesian information criteria resulting from predicting spikes with standard rate maps. The listed effects indicate how BIC changed as a function of each modification to how the rate map is constructed. Negative values indicate improvements. Rows with ‘lag X … int.’ assess interaction effects between each integration approach and the inclusion of a temporal lag.

Abbreviations: DF, degrees of freedom; C.I., confidence interval; int, integration; exp, exponential.

**FIGURE 5:**
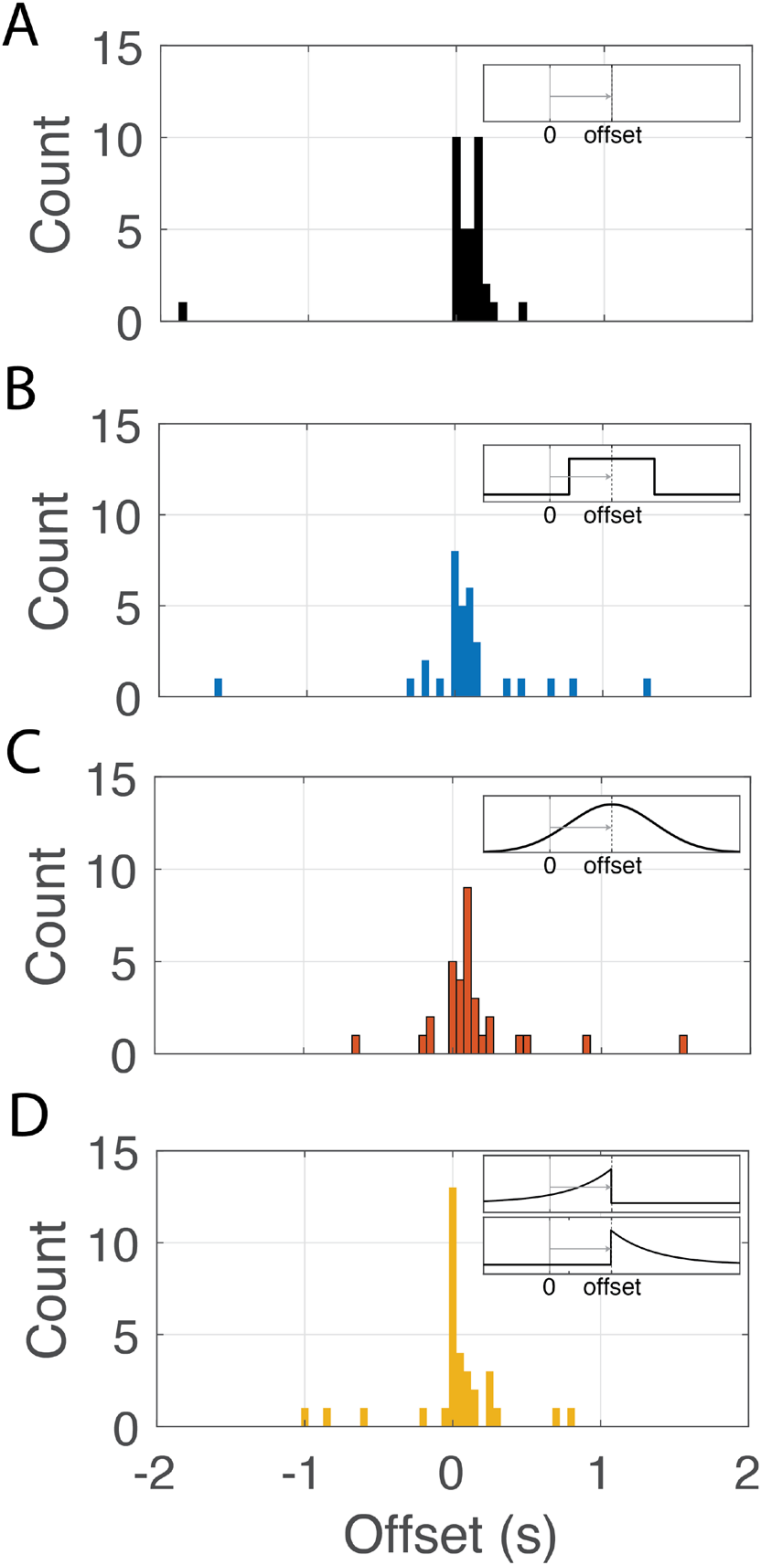
Temporal offsets that create ratemaps with the maximum likelihood of generating the observed spiking are reliably positive. For visibility, the x‐axis was truncated to ±2s. Offsets of outliers are indicated in the caption. (A) The histogram of time offsets over BVCs when no temporal integration is allowed. (B) Temporal offsets when the boxcar integration kernel was used. Points at −7.4 and −5.4 s are not visible. (C) Temporal offsets when a Gaussian integration kernel was used. Points at −7.3 and 3.8 s are not visible. (D) Temporal offsets when an exponential integration kernel was used. A point at −5.8 s is not visible.

The temporal shifts that were selected by the optimization process were reliably positive (Median =84.5ms, $W_{\mathrm{sr}}=566,N=35,Z=4.11,p<0.001$; Figure 5A). However, though the optimized offsets for most BVCs (24 of 35; 69%) were positive, for a subset of the BVCs (10 of 35; 29%) the optimized offset was effectively zero (i.e., within one frame of synchronized to the coincident tracking). One cell (3%) had a negative offset. This finding that the activity of 30% of the BVCs was most predictable without an offset applied could reflect different sub‐populations. With the idea that the subset with positive offsets may reflect a distinct subset of BVCs, we recomputed the median offset for that set after removing the BVCs with negative or zero offset and found it to be 146.0 ms [113.7161.5], approximating one theta cycle. Optimized offset estimates for each cell were reliable across cross‐validations and recordings (Table 1). These results build on those in the prior section by showing that not only does aligning BVC spiking with future position improves spatial tuning, the resulting rate map also provides a more accurate model from which to predict BVC spiking.

### 3.3 Time‐Integration Further Improves BVC Spiking Predictions

Published evidence showing that entorhinal cells encode behavioral variables with a spectrum of timescales, with some neurons tracking rapid changes while others integrate over longer timescales (Chaudhuri‐Vayalambrone et al. 2023), which motivated us to ask whether BVCs similarly encode position with a spectrum of timescales. Central to this line of inquiry is the idea that BVC spiking may not simply encode a specific instantaneous position (albeit at some lag). Rather, BVC spiking may encode states with a broad temporal horizon. To gain perspective on this, for each cell, we optimized a parameter controlling the temporal integration window width. As above, this was done using 90% of each recording and then tested on the remaining 10%.

Critically, the nature of the information being integrated and the method of integration affect how spatial information gets encoded by BVC spiking. Time‐integration kernels with different shapes (e.g., boxcar, Gaussian, or exponential) will result in different predicted spiking dynamics. The spiking of a neuron computing a simple moving average of position estimates would be well approximated by a boxcar integration kernel. Or, the spiking of a neuron computing the average over a population of noisy estimates would be well approximated by a Gaussian integration kernel. Finally, the spiking of a neuron computing temporally‐discounted estimates of future states, used in some reinforcement learning frameworks, would be well approximated by an exponentially decaying integration kernel. To handle this potential dependency, we estimated the temporal integration window separately using each of these kernels (boxcar, Gaussian, and exponential). We then compared the predictability of the BVC spiking as a population under each of the kernels. The full analysis used a two‐by‐four two‐way design, crossing temporal‐offset type [none, optimized] and temporal‐integration type [none, boxcar, Gaussian, exponential]. This permitted us to ask whether there were main effects of including an offset, a time‐integration window, and whether there was an interaction between the two on the ability to predict the observed spiking as quantified by BIC.

**FIGURE 6:**
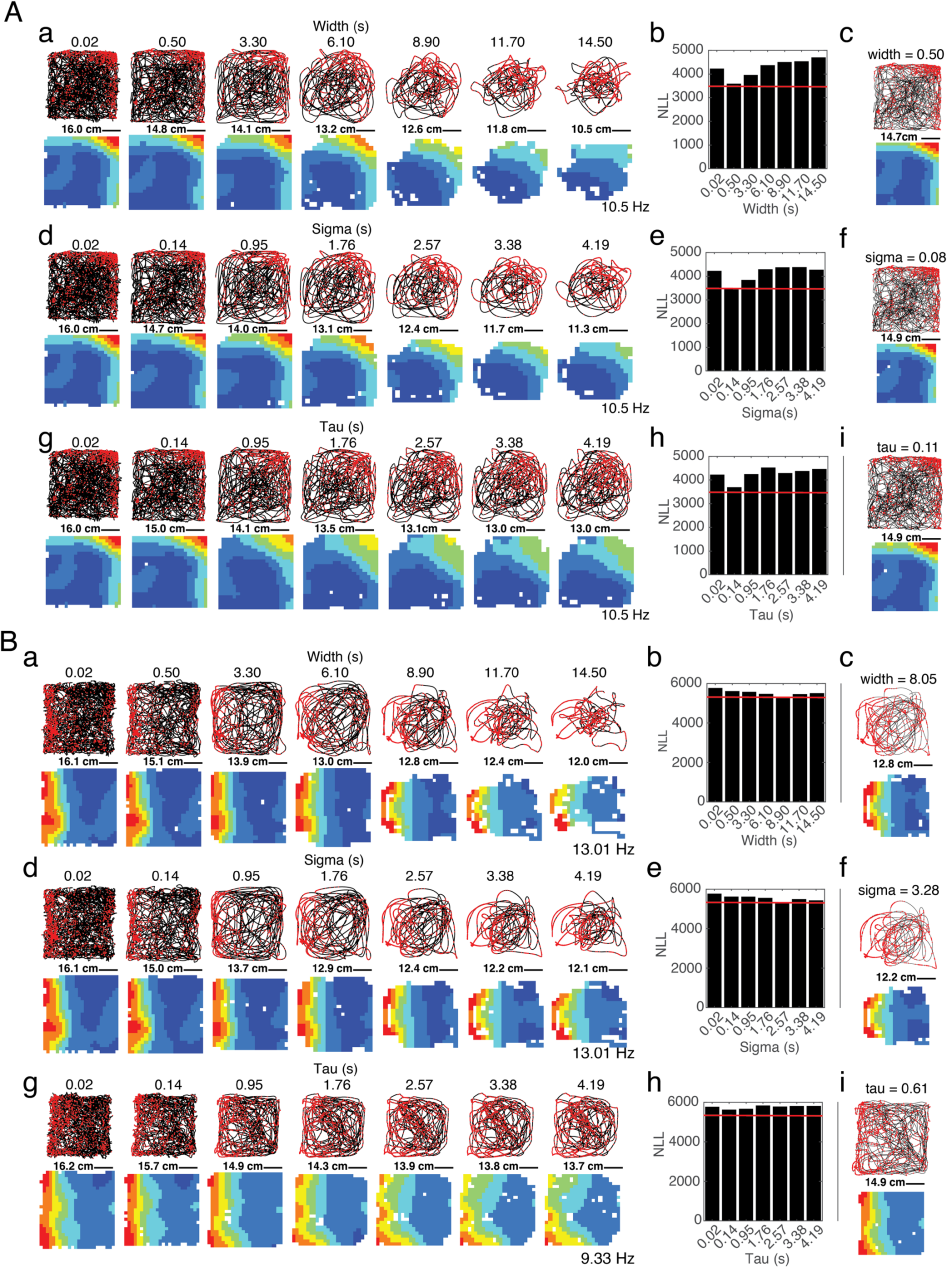
Optimizing a time integration parameter improves the predictability of BVC spiking illustrated by two representative examples (A, B). For each A & B, (a) Top: Example trajectory plots when smoothed with a boxcar time‐integration kernel with seven different widths (as indicated above each plot). The black line shows the time‐integrated position under each parameter value. Red dots mark the estimated position under that integration parameter at the time of each spike. Bottom: The occupancy normalized rate map for the trajectory plot above each panel. Warm colors indicate higher firing rates. All panels are normalized to the same maximum rate, which is indicated at the far right of the row. (b) The negative log likelihood (NLL) for each of the seven sub‐panels shown in (a). Lower is better. The height of the lowest bar across (b), (e) and (h) is marked with a red line. (c) The trajectory (top) and rate map (bottom) for the optimized width computed by the maximum‐likelihood parameterization. (d–f) Same as (a–c) but for varying values of sigma for a Gaussian time‐integration kernel. (g–i) Same as (a–c) but varying values of tau for an exponential time‐integration kernel.

Figure 6 illustrates how manipulating the integration window and type of integration kernel affects the nature of the encoded position estimate. Two example BVCs are shown, one for which the optimized integration window was relatively short (Figure 6A) and one for which the integration window was long (Figure 6B). Trajectory plots and rate maps are shown for wide ranging integration windows for each cell. Temporal integration smooths out small fluctuations in the tracked position at short timescales. At long timescales, the trajectory tracks where the rat lingers.

In all cells of this analysis design, the parameter estimates were highly reliable over cross‐validations and recordings for individual BVCs (Table 1). Comparing BIC values in the two‐way design, we found significant main effects for both temporal offset type (as reported above, see Figure 5B–D) and temporal integration type (*F*(3, 15,250) = 183.27, p<0.001) and found a significant interaction effect (*F*(3, 15,250) = 64.43, p<0.001). Examination of the estimated effects of different levels of temporal offset and integration type on the BIC score, shown in Table 2, reveal that both parameters made rate maps more predictive of spiking.

Improvements in BIC score obtained from adding a temporal integration were substantial across all three variants (integration improved BIC by ∼ 812–940). To test whether one kernel outperformed the others for predicting BVC spiking, we performed post hoc comparisons of BIC under each. The Gaussian kernel outperformed the other two, generating significantly lower BIC scores than the boxcar and exponential kernels (boxcar: est. = −94.6 [−185.0–5.3], *t*(15,432) = 2.08, p<0.05; exponential: est. = −128.1 [−217.5–38.8], *t*(15,432) = 2.8, p<0.005). There was no significant difference between the boxcar and exponential kernels (est. = 33.5 [−55.8, 122.8], *t*(15,432) = 0.73, p=n.s.). For the Gaussian kernel to better explain the variance in BVC spiking than the boxcar or exponential kernels suggests it is a better approximation of what the BVCs encode. The Gaussian kernel approximates a model wherein the BVC spiking encodes the average of noisy estimates of the rat's location.

The significant interaction effect was driven primarily by further improvements in BIC when the temporal offset is combined with the exponential integration approach. Neither of the other combinations resulted in further significant effects on BIC. The specificity of the interaction to the exponential kernel may be a consequence of this kernel placing exponentially greater weighting on the origin (i.e., the point set by the offset) over other time points. In contrast, neither the boxcar nor Gaussian kernels weight the origin so substantially.

**FIGURE 7:**
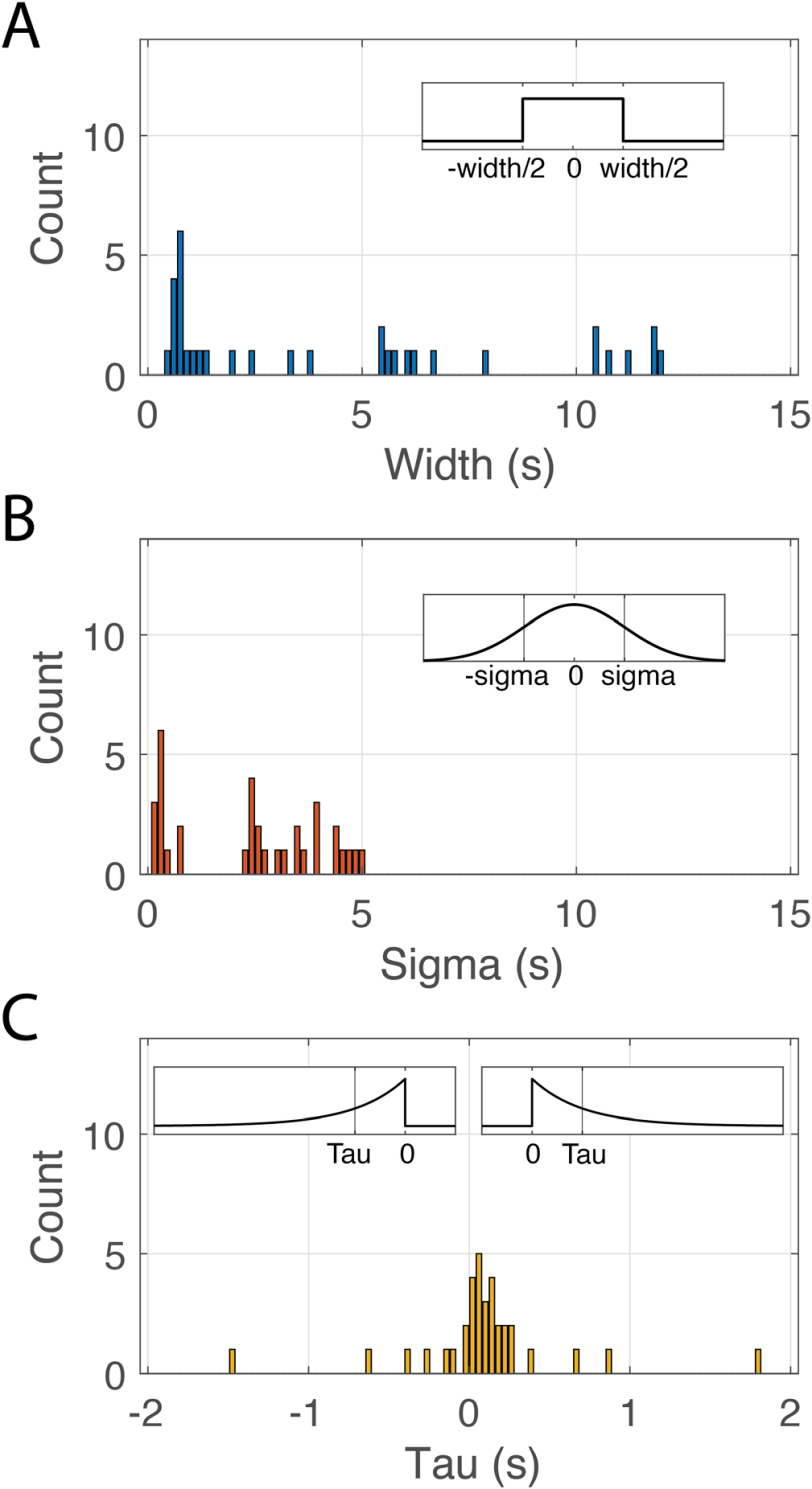
Optimized time integration parameters across kernel types. (A) Histogram of widths used in constructing boxcar kernels. (B) Sigma values used for constructing Gaussian kernels. (C) Tau equivalents of the omega parameter used to construct the exponentially decaying integration kernels. Positive values constructed kernels that decayed into the future, while negative values constructed kernels that decayed into the past.

**FIGURE 8:**
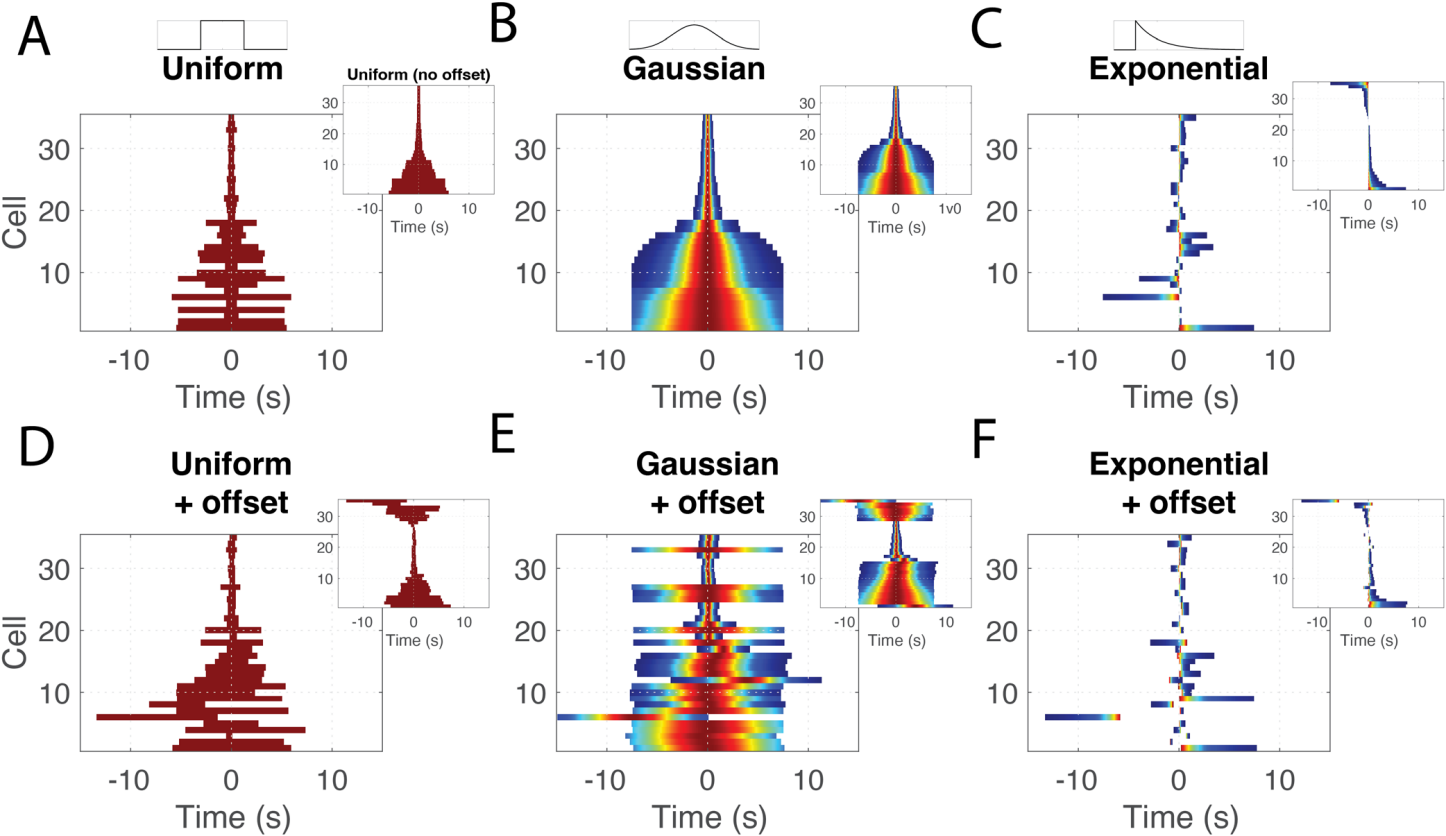
Reconstructed time‐integration kernels across model variants. (A) Horizontal bars show the optimized boxcar kernel integration window width for each cell when no temporal offset was permitted. (B) False color heat maps show Gaussian kernel width for each cell when no temporal offset was permitted. Warmer colors indicate portions of kernel weighted more heavily. (C) Like B but for exponential kernels. (D–F) Same as A–C but for models with temporal offsets. In all large panels, the ordering of cells is the same. The inset panels plot the same data reordered based on the temporal offset and integration parameters for each specific model.

The distributions of time integration kernel sizes are shown in Figure 7. Across all three kernel types, a cluster of the kernels had small magnitudes, indicating that the most predictive ratemaps were generated by limited time integration. However, in each of the cases there was also considerable spread, including values that reflect integration over large temporal windows. The median [95%] sigma for the Gaussian kernels was 2511 ms [795 3307] and a coefficient of variation (CV) of 0.71. The median [95%] width for the boxcar kernels was 2905 ms [1006, 5768] and had a CV of 0.92. The median [95%] tau for the exponential kernels was 246 ms [173, 657] and had a CV of 1.43. The inherent differences across kernels regarding the meaning of these timescale parameters precludes direct comparison across kernels (e.g., the width of the boxcar defines 100% of the window whereas, for the Gaussian kernel, 95.45% of the window is defined by taking two‐sigma from before and after the center point). Yet, what is nonetheless clear is that there is substantial variability (CV >0.5) in the temporal scales that position is encoded with across BVCs. Figure 8 illustrates what these parameters mean in the context of each of the kernels, making apparent the variability in temporal integration width that existed across the optimized kernels.

### 3.4 Time Shift and Integration Windows of BVCs and Non‐BVCs Are Similar

Finally, we asked if the temporal tuning properties were distinct between BVCs and colocalized non‐BVCs. Overall, we found the same qualitative pattern of results for non‐BVCs and found no significant differences in the median optimized parameter values. Precise statistics can be found in Table 3. There was a significant main effect for temporal offset (*F*(1, 25,428) = 156.5; p<0.001) and temporal integration type (*F*(3, 25,428) = 3517.3, p<0.001) and a significant interaction effect (*F*(3, 25,428) = 27.4, p<0.001). Estimated effects on BIC across all levels (see Table 3) show that non‐BVC spiking was more predictable when an optimized temporal offset and integration window were used to model the spatial tuning.

**TABLE 3:** Summary of maximum likelihood results across model types for fits of non‐BVCs.

	BIC	Effect	C.I.	dF	p
No lag, no int.	13,855		$\left[11483,15470\right]$	25,398	
+ time lag		−125.7	$\left[-144.2,-107.1\right]$	25,398	<0.001
+ boxcar int.		−737.5	$\left[-756.1,-718.9\right]$	25,398	<0.001
+ normal int.		−832.6	$\left[-851.1,-814.0\right]$	25,398	<0.001
+ exp. int.		−768.6	$\left[-787.2,-749.9\right]$	25,398	<0.001
lag X boxcar int.		−55.8	$\left[-82.0,-29.6\right]$	25,398	<0.001
lag X normal int.		−95.1	$\left[-106.0,-68.9\right]$	25,398	<0.001
lag X exp. int.		−77.2	$\left[-103.4,-51.0\right]$	25,398	<0.001

*Note:* BIC is the Bayesian information criteria of the standard approach to building ratemaps. Values under ‘effect’ reflect the general linear mixed effect (LME) model estimates of how each modification to how the ratemap is constructed changes the BIC. Negative values indicate improvements in how likely the empirical spiking dynamics were based on the resulting ratemap.

Abbreviations: C.I., confidence interval; DF, degrees of freedom; int, integration.

The optimized temporal offsets of the non‐BVCs, like the BVCs, were reliably positive (median = 122.7 ms [67.1166.7]; $W_{\mathrm{sr}}=1978,N=69,Z=4.6,$ p<0.001). Of the 69 non‐BVCs colocalized with BVCs, the optimized temporal offsets of 51 (73.9%) were greater than 1 frame of behavioral tracking into the future (> 20 ms), 9 (13.0%) were within 1 frame of zero offset, and 9 (13.0%) had offsets of more than 1 frame into the past. Comparing the distributions of optimized offsets between BVCs and colocalized non‐BVCs, we found no significant difference (102.9vs. 122.7 ms; $W_{\mathrm{rs}}=1658,Z=-0.77,$ p=n.s.).

Comparing the different temporal integration kernels, as with the BVCs, we found that the Gaussian kernel yielded position estimates that made the spiking most predictable. BIC values for the Gaussian kernel were significantly lower than those from the boxcar and exponential kernels (Gaussian vs. boxcar: est. = −73.2 [−92.9–53.6], *t*(25,742) = 7.32, p<0.001; Gaussian vs. exponential: est. = −137.4 [−157.0–117.8], *t*(25,742) = 13.73, p<0.001). The boxcar kernel, in turn, produced BIC values that were significantly better than the exponential kernel (est. = −64.2 [−83.8–44.5], *t*(25,742) = 6.4, p<0.001). The finding that the Gaussian temporal integration kernel made the spiking most predictable is consistent with non‐BVCs encoding the mean of a noisy population.

Across the non‐BVCs, there was considerable variability in the scale of the optimized time‐integration windows. For the Gaussian kernel, the median [95% CI] sigma value was 2571 ms [2363, 3674] and a coefficient of variation (CV) of 0.66 (data not shown). For the boxcar kernel, the median [95% CI] width was 5127 ms [3552, 6884] and a CV of 0.77. For the exponential kernel, the median [95% CI] tau (collapsing over forwards and backwards kernels) was 513 ms [298, 873] and a CV of 1.52. As was observed among the BVCs, there is high variability (CV >0.5) in the integration windows across cells irrespective of which kernel type was used to estimate the temporal tuning width. This reflects the diversity of temporal encoding present in the subiculum.

## 4 Discussion

Spatial coding in the subiculum is less well understood than that in other entorhinal‐hippocampal regions such as CA1 and medial entorhinal cortex. See Place et al. (2026); Lee and Lee (2026), and Lever et al. (2026) for subiculum‐centred reviews of hippocampal spatial coding. The present study aimed to better understand the temporal coding properties of boundary vector cells (BVCs) recorded in the rat dorsal subiculum. To this end, we analyzed recordings of BVCs previously described by Lever et al. (2009) using two approaches. In the first, we asked what temporal offset between the rat's position and BVC spike timing yielded rate maps with the maximum apparent spatial tuning. Across BVCs, we found that positive offsets, aligning spikes to future positions, resulted in rate maps with the greatest spatial tuning. In the second approach, we modeled the spiking of each BVC as a function of the rat's position. The models could introduce a temporal offset between the behavioral tracking and spike timing and/or a time‐integrated representation of the position. The BVC spiking was best predicted by models with both the temporal offset and time‐integration of position. The offsets that resulted in the best predictions were reliably positive, aligning spikes with future tracking. The time‐integration windows that generated the best predictions varied widely across cells, ranging from 0.48 s up to 12.0 s. The variability from one cell to the next indicates that there is a multiscale encoding of future states across these BVCs. To establish whether these temporal coding properties are unique to BVCs among subiculum cells, we repeated the same analyses on non‐BVC cells recorded from the same tetrodes. The resulting offsets and parameter estimates were not significantly different between the BVCs and non‐BVCs, indicating that multiscale encoding of future states is likely a general property of the subiculum.

The two analysis approaches used here address related but distinct aspects of neural coding in the subiculum. The first analysis followed an approach that has been used previously to test for multi‐scale temporal coding in the entorhinal cortex (Chaudhuri‐Vayalambrone et al. 2023). The analysis effectively asks, ‘At what temporal offset between the behavioral tracking and the spike timing is the correspondence between the neural activity and position least random?’ This is a computationally efficient analysis that effectively previewed the modeling results of the second approach. Yet, this approach does not address whether the offsets that maximize apparent spatial tuning also describe the spiking of the cells better. Describing the cell spiking is the primary objective of the second approach. The second approach used a *model selection* approach to first ask what free‐parameters significantly improve our ability to predict the neural activity. Subsequent to establishing that a given parameter (e.g., the temporal offset) significantly improved our ability to model the spiking, we could examine the values of that parameter that were most likely to explain the observed spiking (e.g., offset aligned with future positions).

Another strength of the model selection approach is that it allowed us to test between alternate hypotheses for how neurons may encode position. By comparing models that predicted spiking by integrating the position with different kernels, we could test whether the observed spiking was most likely under one form of integration over another. We compared three kernel types: boxcar, Gaussian, and exponential. Each operationalizes a different implicit model for how the neurons integrate the behavioral state. A boxcar integration kernel weights all time points within a window equally. This captures an implicit model wherein the spiking of a neuron is the result of averaging over state estimates spanning a temporal range. The Gaussian integration kernel operationalizes an implicit model, related to the central limit theorem, wherein the spiking of a neuron results from sampling a noisy distribution. Finally, the exponential kernel operationalizes implicit models wherein the activity results from a process that discounts time points further away. Adding time‐integration to the models significantly improved our ability to predict the BVC spiking irrespective of kernel type. However, the Gaussian kernel outperformed the other two. This was the case for both the BVCs and the colocalized non‐BVCs. This is consistent with a mechanism wherein multiscale tuning in the subiculum emerges by each of the cells sampling a noisy distribution.

Given known heterogeneities among BVCs (e.g., trace vs. non‐trace; Poulter et al. (2021)), it is possible that there is variability in information being integrated by the different BVCs analyzed here. Though we lacked the power to subdivide the BVCs functionally, we did find that the spiking of a subset of the BVCs (10 of 35; 29%) was best predicted by models with no temporal offset despite that the spiking of most BVCs (24 of 35; 69%) was best predicted by future positions.

The multiscale temporal coding of behavior documented here is broadly consistent with what has been observed in the reciprocally interconnected entorhinal cortex. Entorhinal grid cells, speed cells, and cells of the lateral entorhinal cortex have all been found to exhibit multiscale encoding of state (Chaudhuri‐Vayalambrone et al. 2023; Dannenberg et al. 2019; Bright et al. 2020; Tsao et al. 2018). Yet, there are also important differences. The multiscale encoding of time in the lateral entorhinal cortex and of speed in the medial entorhinal cortex provide *retrospective* records. Here, however, we find that BVCs are *prospective*. Entorhinal grid cells are also prospective, yet their hexagonal code affords different computations. Multiscale encodings among grid cells can, per established theory (Howard et al. 2014; Shankar et al. 2016), support inferential reasoning about trajectories between distal targets. Multiscale encoding of distance from boundaries could, by a similar reasoning, enable reasoning about the timing of events at a boundary.

What computations might an animal perform at environmental boundaries? One clue is that boundary encounters calibrate medial entorhinal grid cells, acting as an error‐correction signal (Hardcastle et al. 2015). Such correction demands a calibrated estimate of distance to the boundary. A multiscale encoding of distance to a boundary would provide the necessary information for calculating this correction factor. Across a population of cells, there would be graded information about when a boundary was reached. In the moments leading up to and following an interaction with a boundary, there would be sufficient information across the population of BVCs to infer the relative trajectory taken. That estimate could then update the animal's current positional belief or perceived environmental scale.

The future bias of BVCs aligns them with the prospective coding of other spatially tuned neurons of the hippocampal formation (Muller and Kubie 1989; Chaudhuri‐Vayalambrone et al. 2023). Place cells, grid cells, and BVCs all tend to fire 100−200ms before the animal reaches a salient location. This prospective bias is in accord with the hypothesis that the hippocampus encodes a successor representation (Stachenfeld et al. 2017; Momennejad 2020; Geerts et al. 2023). At its core, the successor‐representation view holds that hippocampal activity encodes *what comes next*—not a static map of space or context—conditioned on the current state and habitual transitions. Spike timing dependent plasticity mechanisms of the hippocampus are sufficient to establish successor representations (George et al. 2023). The consistent direction and scale of prospective coding among spatially tuned neurons distributed across the hippocampal formation, including CA1, medial entorhinal cortex, and subiculum, point to the development of the successor representation being a circuit‐wide computation. Interesting directions for future work would be to test if this prospective coding develops as the internal model of an environment develops over familiarization, and would investigate the potential inter‐dependence of different circuit elements (e.g., CA3, CA1, subiculum) for prospective coding.

Multiscale encoding among spatially tuned neurons, including the BVCs examined here, in the context of a successor‐representation view of hippocampal function, may align with temporal discounting (Bos and McClure 2013; McClure et al. 2004; Masuda et al. 2020). Neurons in CA1 encode time and other key parameters necessary for temporal discounting (Salz et al. 2016; Masuda et al. 2020), though it remains to be established that neurons that overtly encode spatial information are key contributors to decision making in the context of delay discounting. Temporal discounting is the idea that future states that will occur after a long delay are encoded more weakly than those that will occur soon but, importantly, are nonetheless encoded. This is, in effect, a way to interpret the multiscale integration windows we observed. Though many BVCs encoded boundaries that will be reached shortly, there were nonetheless BVCs that responded considerably earlier. As such, across the ensemble, there is information encoded about a spectrum of future horizons. This ensemble‐level of encoding is distinct from cellular‐level state encoding, which we failed to find support for when we tested whether exponential integration kernels could predict BVC spiking.

Departing from the successor‐representation view, however, we also observed substantial retrospective coding. In 15% to 30% of our fits, across approaches, we found that the temporal lags that made the empirical spiking most likely were offset into the past. Assuming that this does not reflect measurement error, this is not well accounted for by a standard successor‐representation perspective. Minor modifications could reconcile this mismatch. The Tolman‐Eichenbaum Machine (Whittington et al. 2020), for example, contains elements of the Successor‐representation, including having an imposed bias toward predictive coding. Yet, it also includes associative learning that will form bidirectional associations between states. These associations could, in principle, reactivate prior states given a sensory cue, thereby introducing some retrospective coding.

The present work provided consistent evidence of future‐biased multiscale encoding of position based on the recordings from Lever et al. (2009), but there are several limitations important to acknowledge. One is that the present work did not overtly model the boundary‐oriented nature of the coding itself. Rather, the rate map‐based approach is agnostic to the form of spatial coding. Though this adds a level of generality, the generality runs the risk of missing key elements of the temporal tuning that may only appear in analyses optimized for boundary coding. Part of the reason for not taking that approach here is that accurate modeling of interactions with boundaries would require precise records of the timing of the moments of perceiving a boundary, which is not straightforward to obtain. It is also worth recognizing that there is no ground‐truth for the designations of BVC and non‐BVC. Consequently, cells may have been mislabeled in either direction. The risk of which, as it relates to the current work, is that temporal shifts observed for non‐BVCs may nonetheless reflect BVC coding and that some portion of the heterogeneity in temporal coding observed over BVCs was introduced by inclusion of non‐BVCs. Given the careful comparison of spatial coding over multiple sessions per cell performed by Lever et al. (2009), however, we do not expect this risk to be high, particularly with regard to the possible mis‐classification of non‐BVCs as BVCs. The current findings are unlikely to be merely due to somehow incorrect placement of LEDs upon the rat's head. Inspection of the timeshift curves for the BVCs (Figures 1 and 2) shows the same phenomenon that (Muller and Kubie 1989) observed for CA1 place cells. The rate of degradation of spatial tuning away from the optimal timeshift is not symmetric but rather is slower for shifts that are positive to the optimum (i.e., where spikes align with future positions). “We agree with (Muller and Kubie 1989) view that this asymmetry is incompatible with merely incorrect LED‐placement.”

In conclusion, the current work showed through the use of multiple complementary analyses that BVCs are biased toward encoding future positions and that encoding is most consistent with a time‐integrated version of position. Across cells, we observed variability in the width of the window over which that integration was performed. From these findings, we conclude that BVCs encode a future‐biased spectrum of positions in the rat.

## Funding

This work was supported by National Institutes of Health, R01AG076198; Biotechnology and Biological Sciences Research Council, BB/T014768/1.

## Conflicts of Interest

The authors declare no conflicts of interest.

## Data Availability

The data that support the findings of this study are available from the corresponding author upon reasonable request.
